# The Use of Ginkgo Biloba L. as a Neuroprotective Agent in the Alzheimer’s Disease

**DOI:** 10.3389/fphar.2021.775034

**Published:** 2021-11-04

**Authors:** Anna Nowak, Klaudyna Kojder, Joanna Zielonka-Brzezicka, Jacek Wróbel, Mateusz Bosiacki, Marta Fabiańska, Mariola Wróbel, Joanna Sołek-Pastuszka, Adam Klimowicz

**Affiliations:** ^1^ Department of Cosmetic and Pharmaceutical Chemistry, Pomeranian Medical University in Szczecin, Szczecin, Poland; ^2^ Department of Anesthesiology and Intensive Care, Pomeranian Medical University in Szczecin, Szczecin, Poland; ^3^ Department of Bioengineering, West Pomeranian University of Technology in Szczecin, Szczecin, Poland; ^4^ Department of Functional Diagnostics and Physical Medicine, Pomeranian Medical University in Szczecin, Szczecin, Poland; ^5^ Institute of Philosophy and Cognitive Science, University of Szczecin, Szczecin, Poland; ^6^ Department of Landscape Architecture, West Pomeranian University of Technology in Szczecin, Szczecin, Poland

**Keywords:** Ginkgo biloba L., Alzheheimer’s disease, neurodegenarative disease, EGb 761 extract, dementia

## Abstract

Alzheimer’s disease, a neurodegenerative disease, is one of the most common causes of dementia if elderly people worldwide. Alzheimer’s disease leads to the alienation of individuals and their exclusion from social and professional life. It is characterized mainly by the degradation of memory and disorientation, which occurs as a result of the loss of neuronal structure and function in different brain areas. In recent years, more and more attention has been paid to use in the treatment of natural bioactive compounds that will be effective in neurodegenerative diseases, including Alzheimer’s disease. *G. biloba* L. and its most frequently used standardized extract (EGb 761), have been used for many years in supportive therapy and in the prevention of cognitive disorders. The paper presents an overview of reports on the pathogenesis of Alzheimer’s disease, as well as a summary of the properties of *G. biloba* extract and its effects on the possible pathogenesis of the disease. By exploring more about the pathogenesis of the disease and the benefits of *G. biloba* extract for patients with Alzheimer’s disease, it will be possible to create an individualized therapeutic protocol to optimize the treatment.

## Introduction

**FIGURE 1:**
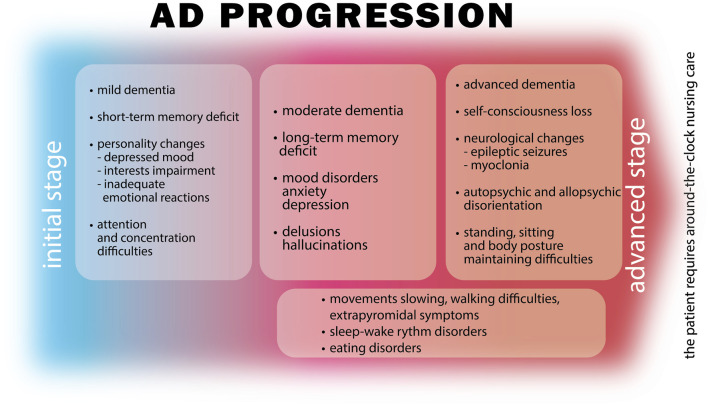
Alzheimer disease progression of symptoms. AD in the initial stage is characterized by short-term memory deficit, personality changes and difficulties in attention or concentration. Progression results in increasing symptoms of memory deficit, mood disorders as well as delusions and hallucinations. Movement difficulties, disorders of sleeping and eating may start occurring. In the advanced stage the disease manifests with self-consciousness loss, auto- and allopsychic disorientation, substantial neurological changes resulting in epileptic seizures, myoclonia or postural stability difficulties. Dementia developing in the course of the disease progresses from mild through moderate to an advanced stage where the patient is getting not capable of independent life.

Alzheimer’s disease (AD) is a chronic neurodegenerative disease that is associated with aging and is one of the most common causes of dementia and social exclusion (Butterfield and Halliwell, 2019; Henstridge et al., 2019; Long and Holtzman, 2019; Singh et al., 2019). AD is responsible for 60–80% of dementia cases and is also their most common cause of death (Katzman, 2008; Alzheimer’s Association Report, 2016). Treatment costs in the USA only were estimated at about $ 1 trillion in 2018 (Patterson, 2018). Despite the prevalence of the disease, the great suffering associated with it and the burden on the patient’s family, the pathogenesis and mechanisms of AD are still not fully understood. Hence, it seems necessary to search for answers concerning the pathogenesis and treatment of AD. The main pathogenetic factors mentioned in literature include: abnormal metabolism of apolipoprotein E, abnormal metabolism–hyperphosphorylation of Tau protein, extracellular accumulation of amyloid beta (Aβ) in the form of senile plaques in the cerebral parenchyma. Moreover, reports indicate the participation of oxidative stress, inflammation, abnormal glial cell function and damage to cholinergic neurons (Trommer et al., 2005; Rhein et al., 2010; Kaur et al., 2015; Nazem et al., 2015; Gargouri et al., 2018; Goschorska et al., 2018; Ma et al., 2019; Singh et al., 2019). In the course of the above phenomena, brain synapses and neurons are damaged, leading to a loss of integrity in neuronal systems, which don’t operate sufficiently in isolation (Henstridge et al., 2019). Some reports also indicate the global effect of cerebral circulation disorders, an improvement of which would have a positive effect on AD treatments (Huang et al., 2020). Damage to such a neurovascular unit would lead to secondary damage to the blood-brain barrier (BBB) (Henstridge et al., 2019). Among the possible causes of dysregulation of homeostasis leading to the development of AD, the possible impact of disorders of the gastrointestinal microbiota is also suggested (Pistollato et al., 2016). There are also extensive studies on the dysfunction of exosomes. They are small vesicles secreted by most cells, including neurons, used for intercellular communication and removal of cell metabolites. They influence the development of neurons and their regeneration. In AD, they are directly related to the transport of amyloid precursors and Tau proteins, which are related to the development of the disease (Xiao et al., 2017). The main cause of this progressive neurodegenerative disorder is the presence of amyloid plaques (AβPs), so-called senile plaques, neurofibrillary tangles (NFTs) and the loss of neurons, especially in the hippocampus and neocortex. These factors accompany the disease from its start at different rate of development. AD is pathomorphologically characterized by granulovacuolar degeneration in the brain, synaptic pathology, rare white matter (white matter refaction) and inflammation (neuroinflammation) (Goschorska et al., 2018; Butterfield and Boyd-Kimball, 2019). The clinical symptoms of AD include: latent episodes of memory impairment and cognitive impairment, psychiatric symptoms, and behavioral disturbances (Nazem et al., 2015; Wang et al., 2016a). Among the spectrum of symptoms also diagnosed as a result of the course and duration of the disease, are communication and behavioral disorders, depression, agitation and dementia (Grewal et al., 2020). The onset of the disease can be tricky, and early symptoms are often confused with fatigue or stress (Gaweł and Potulska-Chromik, 2015). Most often, at the beginning, short-term memory disorders dominate. With disease progression, impairment of the long-term memory develops, and at a deep stage, personal information and identity are lost. Psychiatric manifestations concern speech, orientation, concentration and attention disfunction, also delusions and hallucinations, mood and behavior disorders, depression and psychomotor restlessness. In an advanced stage of AD, neurological changes appear: myoclonus and epileptic seizures affect about 10% of patients, as well as other disorders: extrapyramidal symptoms–slow movements, tilting of the figure, poor facial expressions, difficulties in walking, with a tendency to fall. In the final stage of the disease, difficulties in maintaining a standing and sitting position appear, requiring a lying position, associated with complications such as frequent respiratory infections, thromboembolic complications, decubitus etc. AD is accompanied by eating disorders ranging from malnutrition to polyphagia (in the phase in which the patient is able to eat on his own, associated with memory impairment and not remembering recently taken meals) (Gaweł and Potulska-Chromik, 2015). The natural history of the disease varies individually, and patients live an average of 10 years from diagnosis. AD progression characteristics are presented in Figure 1.

Dementia developed during the course of AD can be mild, moderate or severe. The initial stages of AD include observed changes in recent memory and personality. A depressed mood, weakened interests and guilt are dominant, but also incontinence and inadequate emotional development. In the second stage of the disease, observations, points of view, hallucinations, visual or auditory hallucinations increase. Patients also suffer from disorders of eating, sleep rhythm and wakefulness. At this stage, the patient requires permanent support. In the last stages, muscle tension increases, motor functions become slow, and the patient must sit then lie into the terminal stage, also not auto- or allo-oriented, requiring obligatory permanent 24 h/day assistance. Currently, there is no single drug for treatment of the wide range of AD symptoms, and the development of an effective treatment is a major challenge, so more and more often attention is paid to drugs of natural origin that can be administered alone or in combination with synthetic treatment. Herbal medicines have been in use for over 1,000 years and are one of the most promising sources of new medicines (Belwal et al., 2019). Plants have an abundance of biologically active substances that can eliminate many symptoms of the disease, with a lower risk and occurrence of side effects. One such plant is *G. biloba*, which is characterized by a rich composition of bioactive compounds. The most significant of those are: flavonoids and terpenes (Guo et al., 2020). *G. biloba* leaf preparations are commonly used in the treatment of central nervous system disorders such as Alzheimer’s disease and cognitive deficits (Belwal et al., 2019). Their general action can be described as anti-aggregating (anti-PAF) and nootropic (procognitive). The EGb 761 is often taken by older adults as a supplement to improve memory impairment and the associated cognitive decline. Despite this common usage, the effects and efficacy of *G. biloba* preparations have not been fully investigated, especially in people concomitantly using other anti-AD treatments (Singh et al., 2019).

This review summarizes recent advances in the use of *G. biloba* in AD, as well as the biological effects, molecular mechanisms, toxicity and drug interactions.

## The Factors Influencing AD

**FIGURE 2:**
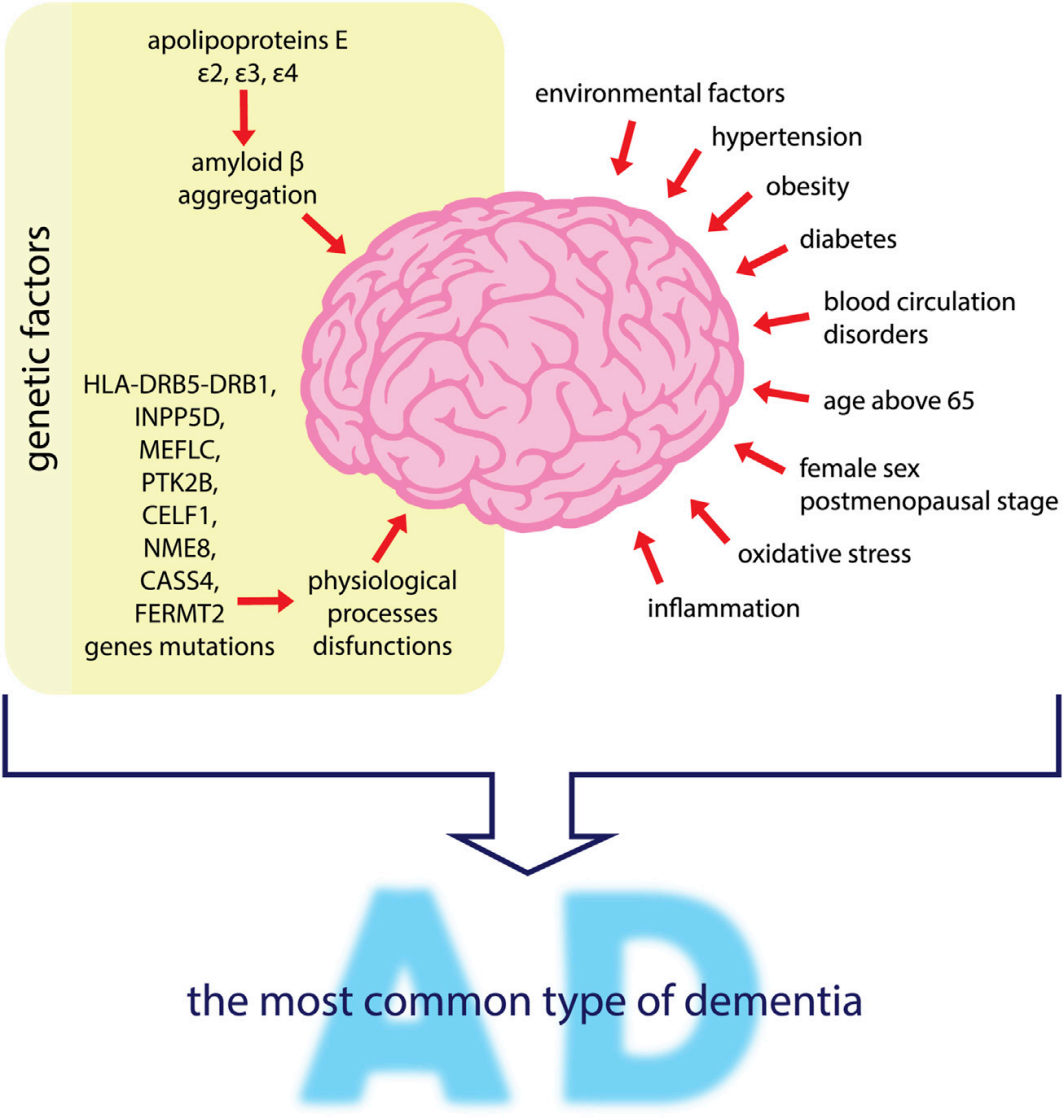
Factors influencing the development of AD–the most common type of dementia. Genetic and environmental factors are mentioned as the main risk factors for developing AD. Apolipoproteins E lead to irregular amyloid *β* aggregation. Mutations of genes such as HLA-DRB5-DRB1, INPP5D, MEFLC, PTK2B, CELF1, NME8, CASS4 and FERMT2 may induce the dysfunctions of brain physiological processes. Other factors such as hypertension, obesity, diabetes, blood circulation disorders, oxidative stress and inflammation, also influence AD progression. People over the age of 65 years, especially postmenopausal women, are more predisposed to AD development.

It has been proven that hypertension, obesity and diabetes have an impact on the development of AD (Liu et al., 2015). The major risk factors for developing AD are genetic and environmental factors, wherein the former concern about 70% of cases and are associated with three variants of apolipoproteins E (APOE), namely *ε*2, *ε*3, and *ε*4 (Ma et al., 2019). APOE *ε*4 is most associated with the increased risk of AD (Sunderland et al., 2004). The effect of the APOE genotype on the risk of AD is likely to depend on the differential effect of APOE on *β* amyloid accumulation in the brain and its blood vessels. Therefore, the response to AD treatment may differ significantly between genotypes (Poirier et al., 1993; Verghese et al., 2011; Ma et al., 2019). Also disorders of lipid homeostasis have a significant impact on the risk of developing AD. Kao et al. (2020) state that abnormal lipid homeostasis could be derived from intestinal microbiota abnormalities and can modify the gut-brain axis. According to the authors, this leads to neuronal signaling pathway disorders, but also to BBB disruption and other changes (such as mitochondrial dysfunction, oxidative stress, and inflammation) at the cellular level that may intensify the course of AD (Kao et al., 2020). Additionally, mutation genes are responsible for creating an environment favorable to the development of AD, such as HLA-DRB5-DRB1 and INPP5D, responsible for the immune response. Also, MEF2C mutations are associated with the immune response and synaptic function, and PTK2B is responsible for cell migration processes and also synaptic function. CELF1, NME8, and CASS4 are involved in shaping the functions of the cytoskeleton and axonal transport. CASS 4 is also involved in the metabolism of Tau and *β* amyloid precursor protein (APP), and FERMT2 in the metabolism of Tau. Mutations of the above genes are detected in AD and may lead to disorders of the physiological processes and development of AD (Karch and Goate, 2015). Age is a key risk factor for developing AD. After the age of 65, the incidence of AD doubles with every 5 years, and the incidence of AD is higher in women than men (Kandiah et al., 2019). In 2018, the number of people with dementia worldwide was estimated at 50 million. By 2030 this number will have increased to 82 million, and by 2050 it will reach 152 million. It is estimated that in high-income countries, the prevalence of dementia is around 5–10% among those aged 65 or over. AD dementia is the most common type of dementia, accounting for 60% of all cases (Hung and Fu, 2017; Ma et al., 2019). Literature reports that the incidence of AD increases significantly in postmenopausal women, reaching 2–3 times that of men. This may suggest a significant protective effect of estrogen in the pathogenesis of AD (Shi et al., 2010). A relationship was also found between disturbances in the sleep-wake rhythm, preceding the preclinical phase of AD development (Musiek et al., 2018). The risk factors mentioned above are listed also in Figure 2 below.

## Treatment of AD

The objectives of AD treatment are to stabilize, slow disease progression, reduce mental and behavioral symptoms, and thereby improve quality of life. To date, no effective drug has been developed to cure or permanently reverse AD pathology. In pharmacotherapy, the aim is now to minimize symptoms and slow down disease progression (Grewal et al., 2020).

### Application of Synthetic Treatments in AD

In clinical practice, the main drugs in AD treatment are synthetic acetylcholinesterase inhibitors (AchEI) such as donezepil, galantamine and rivastigmine, which are first line drugs. They show good effectiveness for patients with mild or moderate symptoms of AD. Tacrine, a drug initially popular for the treatment of AD, is being used less and less because of its side effects. Donepezil–a selective non-competitive acetylcholinesterase inhibitor has a proven efficacy in the treatment of AD. The increase in acetylcholine levels and improvement of cognitive functions in patients with AD has been proven in literature (Huang et al., 2020). The second group of drugs are N-methyl-d-aspartate receptor (NMDAR) antagonists, such as memantine, used for moderate to severe AD symptoms (Birks, 2006; McShane et al., 2006; Kandiah et al., 2019; Ma et al., 2019).

The psychiatric symptoms of AD range from depressive symptoms, most often treated with serotonin reuptake inhibitors (SSRI), which require the addition of new atypical antipsychotic drugs such as olanzapine, quetiapine and risperidone. Conventional antipsychotics are associated with extrapyramidal symptoms more often. The use of both is associated with an increased risk of death in the course of AD (Apostolova, 2016).

### Application of Natural Treatment in AD

In the treatment of AD, natural drugs are being increasingly proposed that could support synthetic pharmacotherapy or, in some cases, replace it. Currently, many plants are being investigated for potential sources of new drugs, especially since multipotential drugs are desirable in the treatment of AD, and those are often drugs of natural origin. Plants provide a wealth of bioactive compounds that may play an important role in the treatment of neurological disorders (Obulesu and Rao, 2011).

### A Combination of Synthetic and Natural Methods of Therapy

Some reports indicate a positive effect of combining natural and synthetic therapy. The simultaneous use of EGb 761 with donepezil (an acetylcholinesterase inhibitor) may give better results in the treatment of AD in terms of cognitive functions than the use of synthetic pharmacotherapy alone (Huang et al., 2020).

## The Use of G. biloba in the Treatment and Prevention of AD

### The History and Description of the Plant

*G. biloba* is a relic and an endemic plant. The genus *Ginkgo* was widespread during the Mesozoic era. Only one species has survived to our times (the so-called “living fossil”). It wasn’t found in a natural state until the middle of the 20th century in the southeast of China. However, it has long been grown at temples in many parts of East Asia as a “sacred tree,” and was planted as an ornamental tree in temperate climate countries. The *Ginkgo* is a gymnosperm dioecious tree. The leaves have a characteristic fan shape and are seasonal (they fall in winter). The leaf blade is leathery, often dissected, with primitive dichotomous veins. The seeds are yellow, on a long stalk surrounded by a fruit-like, fleshy arillus (sarcotesta). The aril contains butyric acid and smells like rancid butter (Hori et al., 2012; Rojas et al., 2016; Belwal et al., 2019). Roasted seeds are edible. In traditional Chinese medicine, *G. biloba* has been used for over 600 years for such diseases as: bronchitis, asthma, renal dysfunction, bladder diseases and as anti-inflammatory substance (Mansoor et al., 2015; Rojas et al., 2016; Strain et al., 2019). The seeds were first used as a medicinal substance by the Yuan dynasty (1,280–1368 CE). Moreover, the first notes of the use of the leaves internally as a medicinal raw material were recorded in the text of Liu Wen-Tai, Ben Cao Pin Hue Jing Yaor in 1,505 (DeFeudis and Drieu, 2000; DeFeudis, 2003; Van Beek and Montoro, 2009). *G. biloba* leaf has long been used primarily in the treatment of brain dysfunction related to brain aging and neurodegenerative dementia (Zimmermann et al., 2002; Fehske et al., 2009).

### Chemical Composition of G. biloba Leaves

**FIGURE 3:**
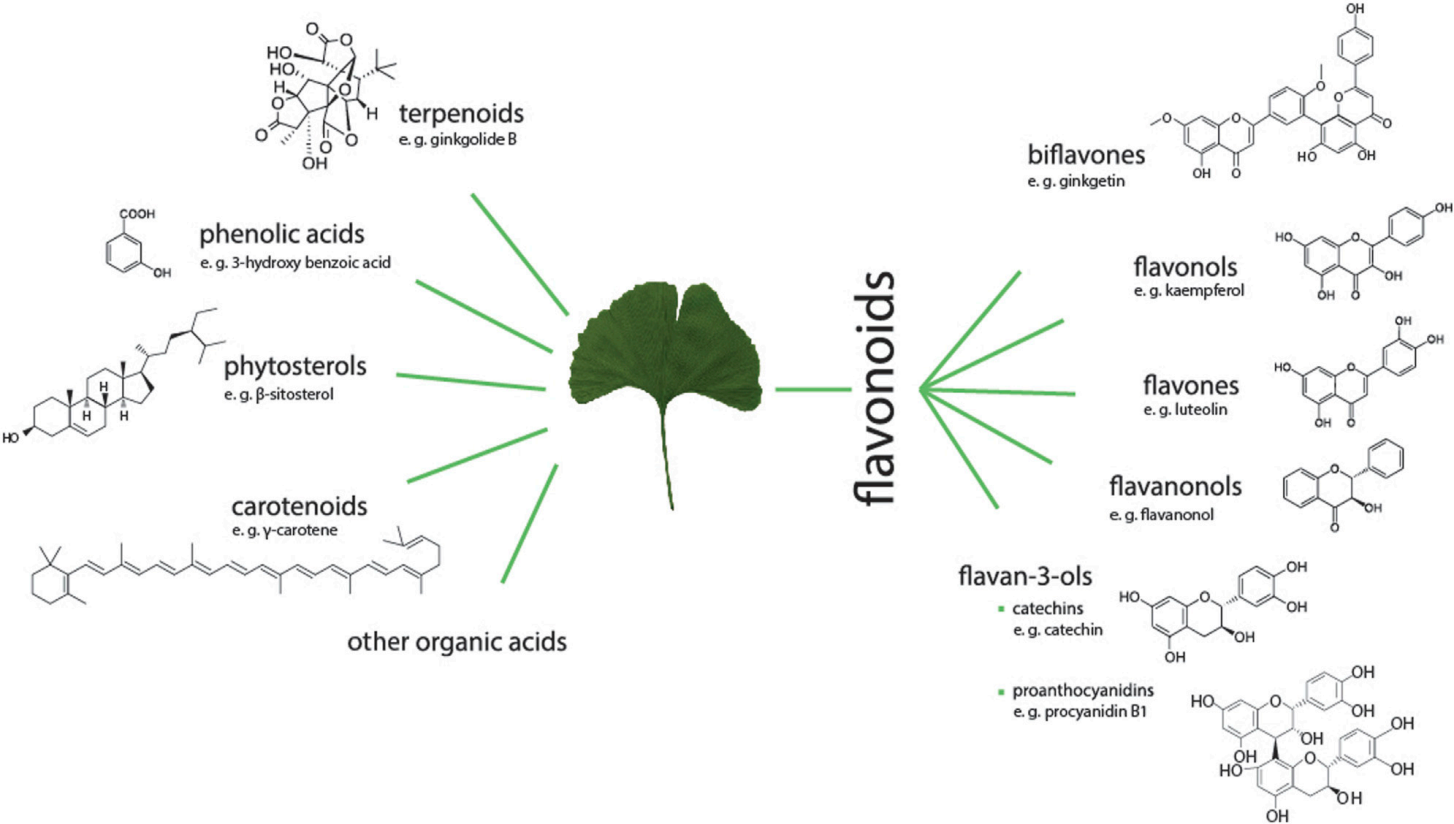
Groups of active compounds contained in GB (G. biloba) leaves with examples of chemical structures. The groups are terpenoids, phenolic acids, phytosterols, carotenoids, organic acids. GB is also a valuable source of a large group of flavonoids including biflavones, flavonols, flavones, flavanonols as well as flavan-3-ols as catechins or proanthocyanidins.

**FIGURE 4:**
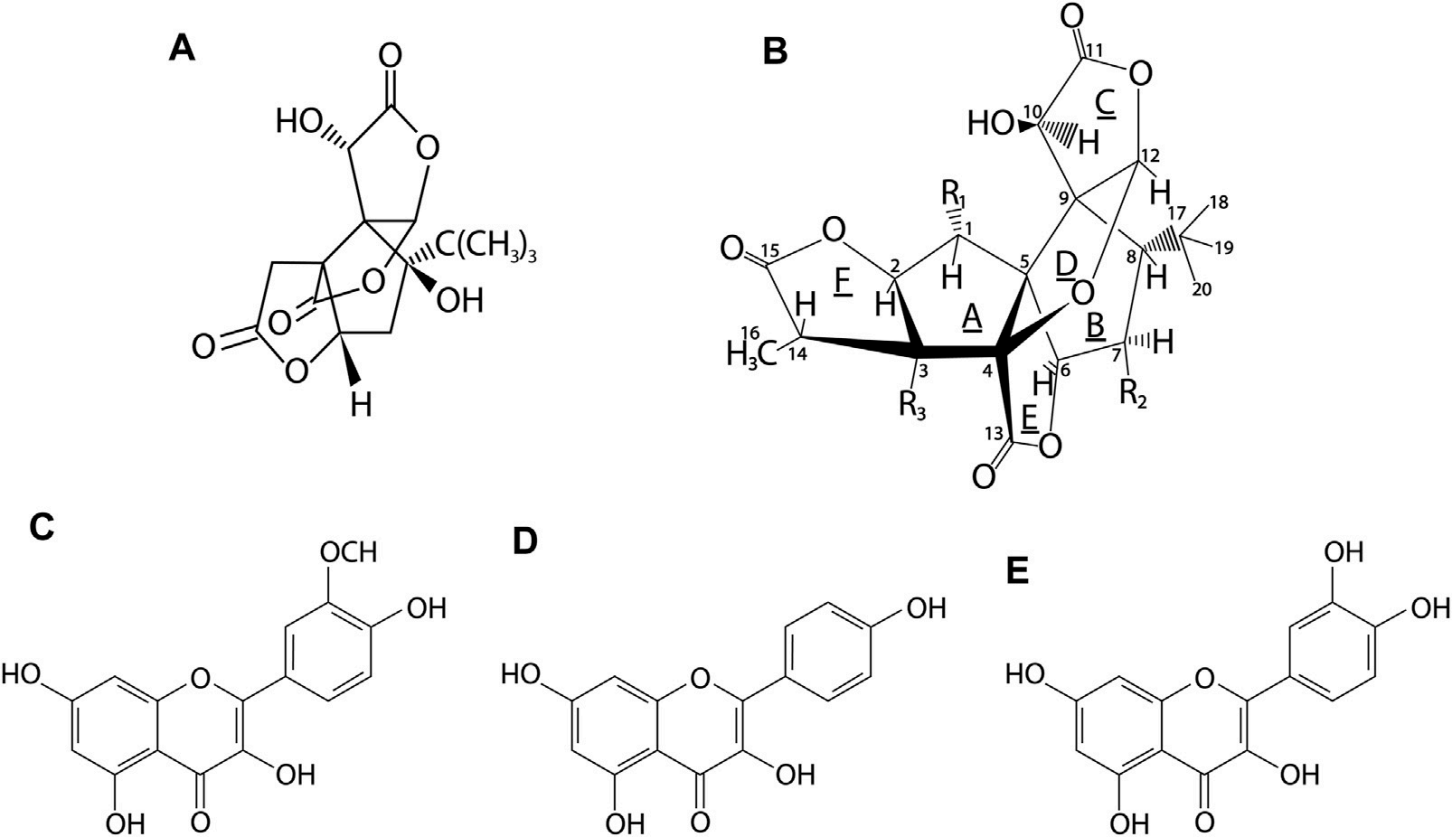
Chemical structures of the major active compounds in G. biloba leaves. **(A)**–bilobalide, **(B)**–ginkgolide, **(C)**–isorhamnetin, **(D)**–kaempferol, **(E)**–quercetin. Ginkgolides contained in GB belong to the group of diterpenes, whereas bilobalide to sesquiterpenes. The main flavonols of GB leaves are kaempferol, quercetin and isorhamnetin (Belwal et al., 2019).

The main components of the leaves of *G. biloba* are flavonoids, constituting the largest group of active compounds represented by various classes of benzo-*γ*-pyrone derivatives (Song et al., 2019). This group includes mainly biflavones: ginkgetin, isoginkgetin, bilobetin, scjadopitizuna, amentoflavone, and the following flavonols: kaempferol, quercetin, isorhamnetin, rutin, myricetin (Van Beek, 2002; Cefali et al., 2019), flavones: luteoin, apigenin and their glycosides and flavanonols (Singh et al., 2008; Kobus et al., 2009; Ren et al., 2019; Chen et al., 2019a). An important group are terpenoids, which include the main diterpenes: ginkgolides A, B, C, J, M, K, L and the main sesquiterpene–bilobalide (Singh et al., 2008; Feng et al., 2019). A potent group are also the derivatives of flavan-3-ol: catechin, epicatechin, epigallocatechin, and gallocatechin. In the leaves there are also proanthocyanidins such as procyanidin, prodelphynidin; biflavon glucosides (ginkgetin, isoginkgetin); phenolic acids: derivatives of benzoic and cinnamic acids; phytosterols such as *β*-sitosterol, stigmasterol, campesterol, dihydrobrassicasterol; and carotenoids such as γ-carotene, α-carotene, lutein as well as organic acids, as shown in Figure 3 and Figure 4 (Singh et al., 2008).

### EGb 761 Extract

**FIGURE 5:**
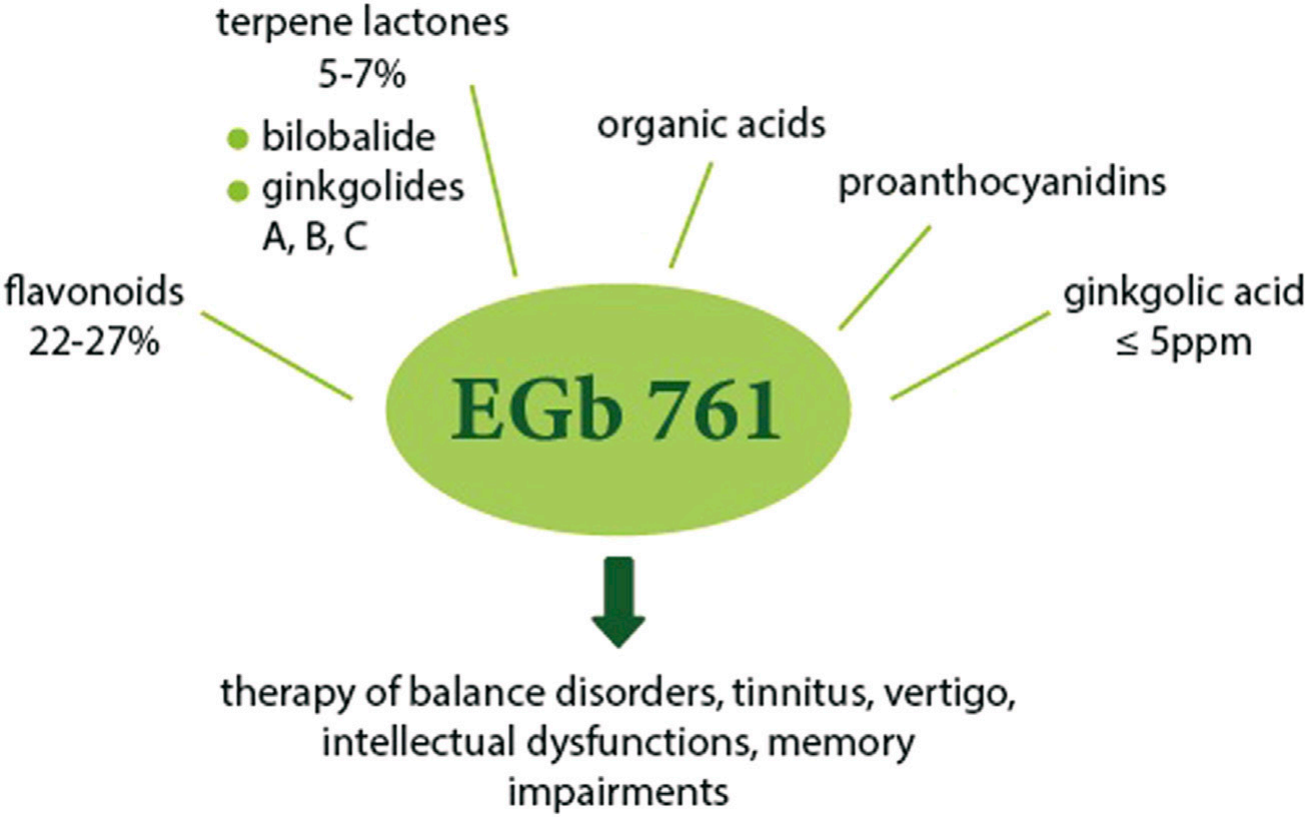
The main components of EGb 761 and their possible uses. EGb 761 is enriched with active substances and contains flavonoids (22–27%), terpene lactones group (5–7%) including bilobalide (2.6–3.2%) and ginkgolides A, B, C (2.8–3.4%), organic acids, proanthocyanidins, with a reduced content of ginkgolic acid (≤5 ppm). The extract could be applied in the treatment of balance disorders, tinnitus, vertigo, intellectual dysfunction, memory impairment and others.

The standardized extract of dried *G. biloba* leaves for pharmaceutical purposes was first obtained and introduced in 1965 in Germany by Dr. Willmar Schwabe III, German physician and pharmacist. The first reports on the therapeutic effect mainly concerned the effect on cerebral and peripheral blood flow disorders and cerebral atherosclerosis and were published in 1965–1966 (DeFeudis, 2003). Then, in 1974 in France, the extract of dried leaves was registered for internal use, receiving the current name–EGb 761. Currently, the standardized EGb 761 extract is one of the most popular herbal supplements. It is in the form of a dry extract made with acetone 60% (w/w) as the extraction solvent. In relation to the original composition of *G. biloba* leaves, EGb 761 is enriched with pharmacologically active ingredients such as flavonoids, terpene lactones, and depleted of any toxic components, mainly ginkgolide acids. The final standardized extract (the components are listed in Figure 5) was adjusted to 22.0–27.0% flavonoids and 5.0–7.0% terpene lactones, consisting of 2.8–3.4% A, B and C ginkgolides and 2.6–3.2% bilobalide, with a ginkgolic acid content less than 5 ppm. EGb 761 has been used in most published clinical and preclinical studies (DeFeudis and Drieu, 2000; Dubey et al., 2004; Lidner et al., 2019; Müller et al., 2019; Piazza et al., 2019). This preparation is used primarily in the treatment of hearing and balance disorders, tinnitus and dizziness resulting from impaired local blood flow, as well as for cognitive dysfunction, in particular age-related memory deficits (Sienkiewicz-Jarosz and Ślusarska, 2003).

### Oxidative Stress in AD

As a result of oxidative stress, the body releases reactive oxygen species (ROS), involved in many disorders, including disorders of the cardiovascular system, tissue damage, DNA damage. All these actions contribute to the development of many neurodegenerative diseases and accelerate aging of the body (Halliwell, 2001; Ahlemeyer and Krieglstein, 2003; Tewari et al., 2017; Nowak et al., 2020). Oxidative stress is a key mechanism in the pathogenesis of AD (Rojas et al., 2016; Zhu et al., 2019). The progressive loss of neuronal cell populations in specific regions of the brain in AD patients is strongly correlated with oxidative stress (Sastre et al., 1998; Smith and Luo, 2003; Rojas et al., 2016). ROS in excess are harmful to all types of cells, including neurons. Such damage in AD can affect all neurons, playing a key role in irreversible cellular dysfunction, ultimately leading to their death. The damage mainly consists of advanced glycation end products (AGEs), lipid peroxidation products and nitration products. Hemoxygenase-1 (HO-1) is one of the most sensitive selective markers of cellular stress response, and in AD both HO-1 and mRNA have been shown to be elevated in the brains of AD patients (Perry et al., 2002). Oxidative stress also accompanies the accumulation of senile plaques in AD, increasing the activity of beta and gamma secretases. The accumulation of Ab plaques supports and intensifies oxidative stress, and together they lead to irreversible changes (Chen et al., 2019a).

### Antioxidant Activity of *G. biloba* vs. AD

One of the methods of protection against oxidative stress is to provide potent exogenous antioxidants. One such is the leaf extract of *G. biloba* (Rojas et al., 2016; Nowak et al., 2017; Tewari et al., 2017; Cefali et al., 2019). The antioxidant effects of EGb 761 are mainly related to its influence on cerebral blood flow, the neurotransmitter system, cellular redox state and the level of nitric oxide (Ahlemeyer and Krieglstein, 2003). EGb 761 works directly by scavenging ROS or increasing the expression of genes encoding antioxidant enzymes. In addition, it demonstrated a protective effect in *in vitro* studies on human brain tissues that had been oxidatively damaged by exposure to hydroxyl (OH·) or superoxide (O_2_^−^·) free radicals produced by Co^60^ irradiation (Siddique et al., 2000). Treatment with EGb 761 extract in rat cerebellar granule cells successfully attenuated the oxidative damage induced by H_2_O_2_/FeSO_4_, which resulted in protection against apoptotic cell death (Wei et al., 2000).

The antioxidant activity in the extract of *G. biloba* is related to its flavonoid components, such as kaempferol and quercetin, which suppressed ROS in the body in both *in vitro* and *in vivo* models (Xin et al., 2000; Smith and Luo, 2003). The flavonoid fraction is responsible for the antioxidant effect through direct ROS scavenging, chelation of pro-oxidative heavy metal ions, and increased expression of antioxidant proteins, such as superoxide dismutase (SOD) and glutathione reductase (GSH) (Rimbach et al., 2001; Sasaki et al., 2002; Ahlemeyer and Krieglstein, 2003; DeFeudis, 2003; Shi et al., 2010). *G. biloba* also reduces lipid peroxidation in the hippocampus, which has been shown in rats (Brdi et al., 2001). The antioxidant effect of flavonoids may result directly from increased activity of the cytochrome P-450 enzyme system, which reduces the formation of ROS and inhibits the release of peroxide anions (Bodalski and Karłowicz-Bodalska, 2006). Other components that determine the antioxidant effect of *G. biloba* is the group of ginkgolids, characterized by an inhibitory effect on the peroxidation of lipids, being antagonists platelet aggregation factor (PAF)—a factor initiating the formation of ROS. Ginkgolide B, which plays the main role as scavenger and ROS inhibitor, turned out to be particularly active (Bodalski and Karłowicz-Bodalska, 2006). It reduces the activity of constitutive and inducible nitric oxide synthesis (cNOS and iNOS) and the permeability of the blood-brain barrier, which may be of particular importance in the prevention of cerebral edema (Sienkiewicz-Jarosz and Ślusarska, 2003; Smith and Luo, 2003). Ginkgolide B, decreased the level of reactive oxygen and nitrogen species (ROS/RNS) in human IMR-32 and SH-SY5Y neuroblastoma cells (Kaur et al., 2015). The specific structure of the polyphenols contained in EGb 761 allows them to capture superoxide anions, hydroxyl radicals and peroxide radicals (Ahlemeyer and Krieglstein, 2003; Shi et al., 2010).

EGb 761 extract blocks A*β*-induced cell apoptosis as a consequence of oxidative stress (Shi et al., 2010). Treatment of mice with EGb 761 for a period of 2 weeks at a dose of 100 mg/kg resulted in a decrease in apoptosis of lymphocytes in the spleen of young (3 months old) and older (24 months old) animals (Schindowski et al., 2001). In the anti-apoptotic effect, apart from ROS scavenging and inhibition of lipid peroxidation, the inhibition of caspase 3 plays a major role (Sienkiewicz-Jarosz and Ślusarska, 2003). Luo et al. (2002) investigating the effect of EGb 761 on a culture of neuroblastoma cells with mutations typical for AD, showed a reduction in the number of cells undergoing apoptosis by inhibiting caspase 3. The apoptosis-reducing effect is most often ascribed to bilobalide (Ahlemeyer et al., 2001). This component also improves angiogenesis through the endothelial nitric oxide synthesis pathway (Zhang et al., 2018).

### Mitochondrial Changes in AD

In the pathogenesis of AD, more and more attention has been focused on mitochondrial dysfunction. Abnormalities in the functioning of mitochondria are related to the pathological changes observed in AD (Eckert et al., 2003a; Hauptmann et al., 2006; Kapogiannis and Mattson, 2011). The impaired function of these organelles reduces the production of ATP, leading to cell apoptosis (Holper et al., 2018). Mitochondria, and in particular mitochondrial DNA (mtDNA), are the primary targets of ROS interactions. MtDNA accumulating in mammalian tissues undergoes at least 10 times more damage from ROS than nuclear DNA. One reason for this is the lack of protective histones and effective repair systems in mtDNA, which results in greater susceptibility to various types of mutations or oxidative damage. The change in mitochondrial function is caused mainly by: 1) aging of the body, 2) overproduction of ROS, 3) overload of Ca^2+^ ions due to their increased uptake from the cytoplasm. These factors lead mainly to a change in the potential of the mitochondrial membrane and its greater permeability, as well as a change in cellular respiratory activity, resulting in an inhibition of mitochondrial respiration (Cavallucci et al., 2013; Wang et al., 2016a). Cell aging also causes an increase in edema and changes in the structure of the mitochondria. Such damage is manifested in the vacuolization of the matrix, damage to the mitochondrial membrane, shortening of the mitochondrial crest. Mitochondrial dysfunction is also associated with decreased activity of mitochondrial enzymes (Sastre et al., 1998; Müller et al., 2019). Such changes of mitochondria in the brain have been observed in the earlier and later stages of AD, which has been classified as one of the main causes of this disease (Müller et al., 2019). The mitochondrial cascade hypothesis for AD states that dysfunction is an important common pathomechanism for the entire spectrum of age-related memory disorders. The most important assumption of this hypothesis probably concerns the very early stages of mitochondrial dysfunction caused by oxidative stress causing aging and a slight increase in Aβ levels (Müller et al., 2019). The aging process of cells is associated with an excessive production of nitric oxide (NO), which reacts with mitochondrial complex IV, causing the inhibition of the respiratory chain. Complex IV may temporarily increase leakage of the superoxide anion from the electron transport chain. The resulting peroxide can then react with NO to form peroxynitrite, which is especially dangerous to mitochondrial enzymes. Consequently, this leads to a reduction in ATP formation and a reduction in the potential of the mitochondrial membrane (Pacher et al., 2007). The most visible defect in the mitochondrial electron transport system in AD is the deficiency of cytochrome C oxidase activity (complex IV), which has been demonstrated in postmortem tissues of human and animal brains. The decrease in ATP synthesis by complex V may initiate apoptosis (Rhein et al., 2010).

### *G. biloba* Vs Mitochondrial Function

EGb 761 has a broad pharmacological activity mainly due to its ROS scavenging properties and its direct protective effect on mitochondria (Abdel-Kader et al., 2007; Li et al., 2019). EGb 761 protects neurons from the β-toxicity that causes mitochondrial dysfunction as well as respiratory chain complex deficiencies and apoptosis (Xie et al., 2013; Wan et al., 2014; Liu et al., 2015). EGb 761 has an effect on the plasticity of neurons, demonstrating long-term synaptic enhancement (LPT), which was observed in the hippocampus of older mice compared to younger animals (Williams et al., 2004; Müller et al., 2019). When used, it reduces oxidative stress, improving mitochondrial respiration (Müller et al., 2019) and protects cells from NO-induced neurotoxicity by reducing the increase in caspase-9 activity that activates caspase-3, leading to cell apoptosis (Massieu et al., 2004; Eckert et al., 2005).

The effectiveness of *G. biloba* against complexes I, IV and V of the mitochondrial respiratory chain was observed against senescent cells obtained from two age groups of mice with induced nitrosation stress. The animals were treated with EGb 761 for a period of 14 days at a dose of 100 mg/kg. The effect was only visible in relation to cells obtained from older animals, which proved that the extract is effective against senescent cells (Abdel-Kader et al., 2007). Under the influence of EGb 761, the levels of ROS in the brains of the older mice were also reduced by increasing the production of ATP in neurons. The long-term influence of EGb 761 on mitochondria is manifested in the regulation of energy metabolism deficits caused by A*β* (Eckert, 2012). EGb 761 protects against the oxidation of mitochondrial glutathione and the increase of production of peroxide by these organelles. The antioxidant effect is related to removal of the superoxide anion and the hydroxyl radical, which consequently prevents lipid peroxidation in the mitochondrial membranes. EGb 761 reduces the production of ROS in the mitochondria and protects the mitochondrial complexes of the respiratory chain and increases the availability of ATP (Eckert et al., 2003b; Abdel-Kader et al., 2007; Baliutyte et al., 2014). The components of EGb 761 that play a key role in reducing ROS are flavonoids, characterized by a high antioxidant potential (Schaffer et al., 2012; Müller et al., 2019). These substances are mainly attributed a protective effect on mtDNA (Sastre et al., 1998). Bilobalid, rutin, quercetin and quercitrin inhibit the hypoxia-induced decrease in ATP content, stimulating the rate of mitochondrial respiration (Trumbeckaite et al., 2007). Terpenes such as: ginkgolide B and J also prevent A*β*-induced mitochondrial dysfunction while reducing intracellular ROS production (Shi et al., 2009; Eckert, 2012). Abdel-Kadar et al. (2007) confirm that both the flavonoid fractions and the terpene fractions present in EGb 761 show a protective effect on these organelles by investigating the effect of the individual fractions on the protection of the mitochondrial membrane potential of PC12 cells treated with SNP (sodium nitroprusside). Considering the fact that the flavonoid fraction constitutes 24% of the extract and the terpene fraction 6%, each of the ingredients used showed a protective effect. The most effective fractions were ginkgolide J and the entire flavonoid fraction, which stabilized the potential of the mitochondrial membrane of the brain cells by 109 and 106%, respectively. The lowest protective effect was shown by ginkgolide A (Abdel-Kader et al., 2007). Bilobalid increased cellular glucose uptake, regulated the process of its oxidation, prevented decoupling of the individual stages of oxygen phosphorylation, which in turn prevented a decrease in ATP levels and its further consequences, such as ionic disturbances or mitochondrial dysfunction (Sienkiewicz-Jarosz and Ślusarska, 2003). A consequence of the protective effect of EGb 761 on the mitochondrial respiratory chain complex is the improvement in neuronal functions previously impaired as a result of aging, hypoxia, hypoglycemia or an increase in A*β* (Rhein et al., 2010; Eckert, 2012; Baliutyte et al., 2014; Wan et al., 2014; Müller et al., 2019).

### Neuroprotective Effect of G. biloba

Loss of neurons is a factor of cognitive deficit in AD (Aguirre-Rueda et al., 2015; Akhter et al., 2018). EGb 761 extract has a neuroprotective effect, including: inhibiting the inflammatory process (Wan et al., 2016), inhibiting apoptosis (Liu et al., 2015), lowering the level of amyloid precursor protein (APP) (Augustin et al., 2009), including A*β* (Tchantchou et al., 2007), while increasing cell proliferation in the hippocampus (Tchantchou et al., 2007). The exact mechanism of *G. biloba* neuroprotective effect at the cellular level, is not fully understood, but include: 1) scavenging of free radicals, 2) improving mitochondrial function, 3) reducing blood viscosity, 4) serotonin levels modulation in various regions of the brain, 5) increasing the level of dopamine in the prefrontal cortex (Shi et al., 2010; Kandiah et al., 2019). EGb 761 extract administered intraperitoneally to mice (20, 50 and 100 mg/kg/day for a period of 7 days) before or after treatment with 1-methyl-4-phenyl-1,2,3,6-tetrahydropyridine (MPTP), protected the nigrostriatal dopaminergic pathway (nigrostriatal dopaminergic) with a decrease in monoamine oxidase (MAO) activity in the brain (Yang et al., 2013). Administration of EGb 761 for 7 days in the middle cerebral artery occlusion (MCAO)—induced focal cerebral damage I/R model in rats resulted in an improvement in neurological deficits by reduction of the content of malondialdehyde (MDA) and pro-inflammatory cytokinins tumor necrosis factor (TNFα) and interleukin 1*β* (IL-1β) while increasing the level of anti-inflammatory cytokinin interleukin 10 (IL-10). This study also noted the activity of SOD and myeloperoxidase (MPO) (Yang et al., 2013). Hao et al. (2016) demonstrated the neuroprotective effect of EGb 761 when co-administered with bone marrow derived mesenchymal stem cells (BMSCs) in encephalomyelitis (EAE). Consequently, combination therapy with BMSCs and EGb 761 showed an synergistic effect in animals with experimental autoimmune encephalomyelitis through inhibition of pro-inflammatory cytokines, demyelination and protection of axons and neurons.

### Microglia Function in AD

Microglial cells play an important role in the development and prevention of many diseases of the central nervous system, such as AD (Doens and Fernández, 2014). Microglia are non-neuronal cells of the central nervous system that control homeostasis and are involved in the immune response. Microglia activation, which causes tissue damage, is associated with an increase in the level of immune function factors. Microglial cells are extremely sensitive to brain damage (Ziaja, 1995). They can participate in disease processes by secreting A*β*. Reactive microglia can influence the development of AD disease through increased secretion of interleukin (IL-1), which in turn causes increased expression of APP and *α*-antichymotrypsin, which are components of senile plaques (Ziaja, 1995). Inflammation is of key importance in the development of AD, as confirmed by epidemiological studies on the inhibition of progression of AD in people taking anti-inflammatory drugs (Goschorska et al., 2018). Nerve tissue inflammation is a key factor in AD, increasing microglia activation. On one hand, microglia causes the loss of neurons through the release of pro-inflammatory cytokines, and on the other, they have beneficial effects in the form of neuronal repair (Tang and Le, 2016). It seems reasonable to say that the maintenance of nerve cell homeostasis depends on a certain balance between pro and anti-inflammatory activation, in which microglial cells participate (Goschorska et al., 2018).

Proper microglia function is also related to the brain’s ability to maintain proteostasis. The reason for this not happening may be a weakened primary immunity and a lack of broad scope for the development of secondary immunity in the brain tissue compared to other tissues. As a consequence, they can damage the delicate mechanism of central nervous system homeostasis. Supporting the nervous system in its natural ability to utilize the beta amyloid and plaques without exacerbating inflammatory features is currently one of the lines of work in the treatment of AD (Shi et al., 2009). Perhaps natural pharmacotherapy has such properties, but that requires more extensive research.

### G. biloba vs. Microglia

**FIGURE 6:**
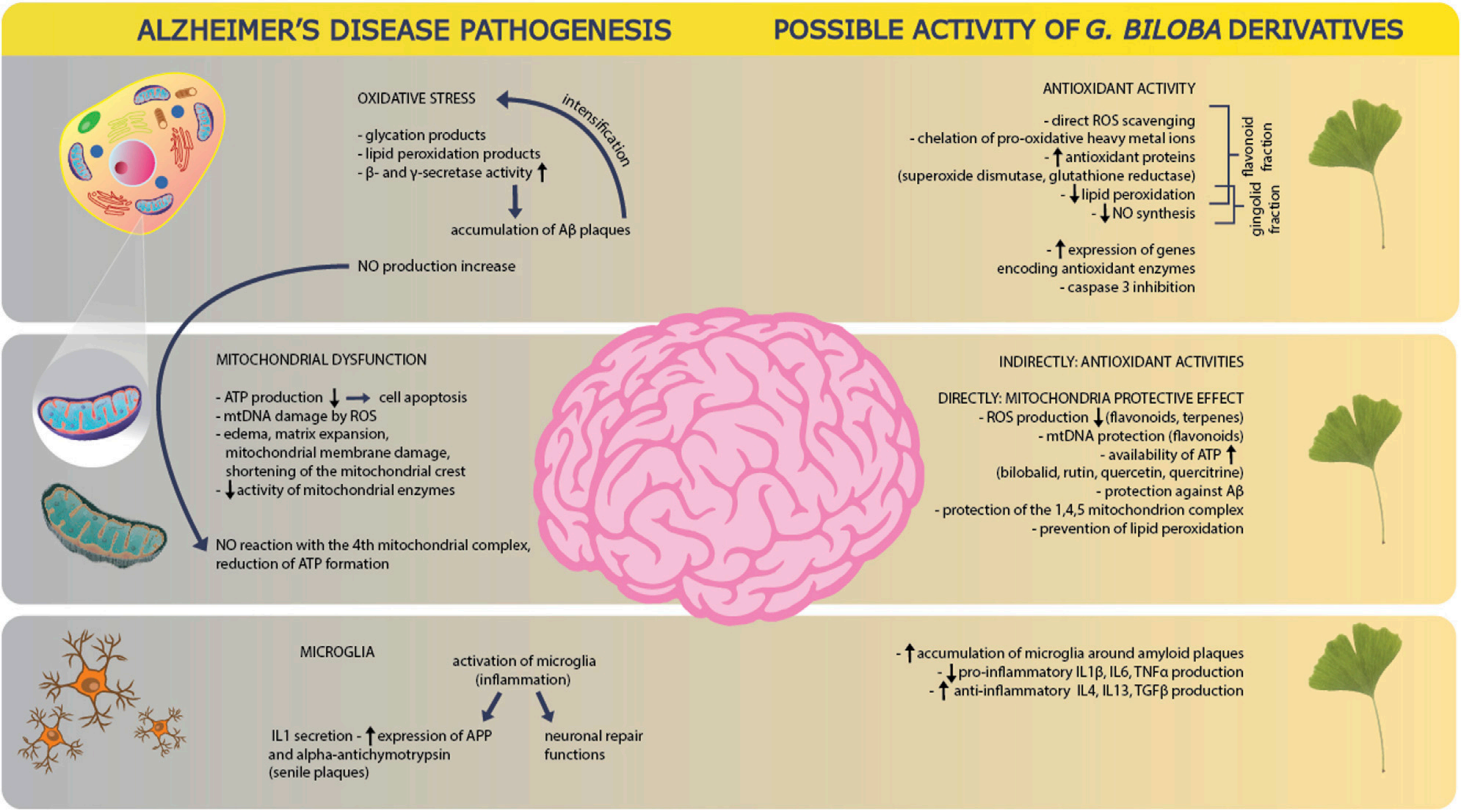
Alzheimer’s disease pathogenesis and possible activity of *Ginkgo biloba* derivatives. In the pathogenesis of AD, one of the main causes is oxidative stress, as a result of which the products of glycation and lipid peroxidation are formed. It also leads to an increase in the activity of beta and gamma secretases, which stimulate platelet activation, which in turn increases the oxidative stress itself. There is also an increase in NO production. GB derivatives counteract these phenomena by direct trapping of ROS, chelation of active metal ions, delivery of antioxidant proteins, reduction of lipid peroxidation and NO synthesis–mainly with the help of the flavonoid and ginkgolide fractions. The derivatives also reduce oxidative stress by increasing the expression of antioxidant enzyme genes and inhibiting caspase 3. Another element in the pathogenesis of AD is mitochondrial dysfunction. In the disease, ATP production decreases, which accelerates cell apoptosis. The damaging effect of ROS on mitochondrial DNA is also reported. The pathogenesis involves swelling, expansion of the mitochondrial matrix and damage to the mitochondrial membranes, as well as the shortening of mitochondrial crests, which is accompanied by a decrease in the activity of mitochondrial enzymes. NO produced through oxidative stress, reacts with the fourth mitochondrial complex and leads to a reduction in ATP production. GB derivatives counteract the mitochondrial dysfunction indirectly by limiting oxidative stress in general. Directly GB derivates react by decreasing ROS production, protection of mitochondrial DNA, supporting ATP availability, protection against platelet production and accumulation as well as protection of mitochondrial complexes themselves: 1,4,5. They also reduce lipid peroxidation. At the level of microglial function in AD, these cells of the immune system are activated due to inflammation, which leads to an increase in pro-inflammatory cytokines: IL1 and expression of APP as well as alpha antichymotrypsin. On the other hand, microglia activation also triggers neuronal repair mechanisms. GB derivatives support the accumulation of microglia around amyloid plaques, and also lead to a reduction in the production of pro-inflammatory cytokines (IL1β, IL6, TNFα) and to increase the production of anti-inflammatory cytokines (IL4, IL13, TGFβ).

Inflammatory responses resulting from A*β* deposition in the cerebral parenchyma are mediated by numerous mechanisms, including microglia activation and inflammasome activation (Thériault et al., 2015). An inflammatory process mediated by microglia in the central nervous system plays an important role in the loss of neurons in various neurodegenerative diseases, including AD (Wang et al., 2015). The use of EGb 761 significantly increases the microglia around amyloid plaques. Microglia releases cytotoxic mediators such as IL-1β, IL-6 and TNFα, causing pro-inflammatory reactions, however, anti-inflammatory cytokines such as IL-4, IL-13 and Arg-1 are also secreted in microglia activation. The use of EGb 761 significantly reduces the above-mentioned cytotoxic mediators, which were found in the brains of animals supplemented with EGb 761. On the other hand, the level of anti-inflammatory cytokines such as IL-4, IL-13 and TGFβ was significantly increased. Upregulation of anti-inflammatory factors with simultaneous downregulation of pro-inflammatory cytokines suggests that EGb 761 may be sensitive to the microglia phenotype. This hypothesis is adopted by Wan et al. confirming the reduction of the iNOS marker of the M1 phenotype and the increase of the Arg-1 marker of the M2 phenotype in a study on transgenic aPP/PS1 mice, supplemented with EGb 761 for a period of 6 months iNOS and Arg-1 are considered markers of the M1 and M2 phenotypes, respectively. M1 marker iNOS was decreased and the M2 marker Arg-1 was increased in chronic EGb 761 treated mice (Wang et al., 2016b). More evidence for the reduction of the pro-inflammatory response by EGb 761 is the inhibition of microglial inflammatory responses using BV2 microglial cell lines, which, assessed by qPCR, showed a significant increase in pro-inflammatory cytokines TNFα, IL-1β and IL-6 compared to control cells. Moreover, supplementation with EGb 761 increased the levels of mRNA, macrophage inflammatory protein-1 alpha (MIP-1α) and monocyte chemo attractant protein-1 (MCP-1) (Wang et al., 2016b). EGb 761 extract strongly inhibits LPS-induced prostaglandin E2 (PGE 2) production mediated by cyclooxygenase 2 (COX-2) and other pro-inflammatory mediators such as TNF-α, IL-6 and IL-1β in studies in rats (Gargouri et al., 2018). Long-term binding of EGb 761 can reduce the pathology of Aβ by acting to inhibit *β*-secretase activity and Aβ aggregation. A positive effect of EGb 761 extract was also shown in reducing chronic inflammation of the colon in mice, inhibiting macrophage activation and reducing inflammation by lowering inflammatory markers such as: iNOS, COX-2, TNF-α. Moreover, it was shown that the EGb 761 extract reduced the number of T cells such as CD4 +/CD25-/Foxp3 in the colon (Kotakadi et al., 2008). EGb 761 reduced the activity of cyclooxygenase 1 (COX-1) and cyclooxygenase 2 (COX-2), which influenced the selective inhibition of thromboxane A2 mediated by COX-1 in platelets, and the production of prostaglandin I2 (PGI 2) mediated by COX-2 in endothelial cells (Kudolo et al., 2002). Free cholesterol may be involved in the production of A*β* protein and APP (Howland et al., 1998; Galbete et al., 2000; Yao et al., 2001; Yao and Papadopoulos, 2002; Yao et al., 2004). This thesis was confirmed by Yao et al. (2004), who demonstrated a simultaneous reduction of free cholesterol level and a decrease in the production of APP and Aβ in the brain of older rats after using EGb 761—Figure 6.

### Memory and Cognition in AD

Cognitive decline is recognized as one of the main symptoms of AD. In the elderly, the prefrontal cortex plays a key role in cognitive function, where prefrontal dopaminergic innervation decreases with age. This results in a decrease in the control of cognitive functions, i.e., cognitive flexibility, goal retention, inhibition of habitual or impulsive reactions and prospective memory (Ruge et al., 2013; Sreenivasan et al., 2014; Beck et al., 2016; Zhu et al., 2019).

### The Role of G. biloba in Improving Cognitive Functions

The primary clinical application of EGb 761 includes improvement of cerebral and peripheral circulation as well as a positive effect on neurosensory dysfunction (Yu et al., 2018; Singh et al., 2019; Kandiah et al., 2019; Liang et al., 2020). Numerous studies have confirmed the beneficial effects of EGb 761 and other standardized *G. biloba* leaf extracts on cognition and age-related problems with memory and concentration. EGb 761 showed a beneficial effects in both healthy and AD subjects (Table 1). The main reasons for the improvement in memory and cognitive functions after the use of *G. biloba* include: increased blood flow in the brain, protective effect against peroxidation of brain lipids, easier utilization of oxygen and glucose by brain cells, reduction of amyloid plaque deposition (Wan et al., 2016), lowering the level of A*β* oligomers (Tchantchou et al., 2007) and APP levels (Augustin et al., 2009). *G. biloba* affects several neurotransmitter pathways, as demonstrated by studies in rats administered EGb 761 for 14 days. The extract also reduced stress-induced excessive corticosterone secretion by reducing the number of peripheral benzodiazepine adrenal receptors (Marcilhac et al., 1998). EGb 761 can effectively reduce the activity of MAO, as well as increasing the level of dopamine, especially in the prefrontal cortex (Rojas et al., 2004). The dopamine-boosting effect is probably based on a mild inhibition of the norepinephrine transporter, a protein mediating the synaptic uptake of the dopamine hormone (Beck et al., 2016). An increase in serotonin levels was also observed in the prefrontal cortex and hippocampus of mice that consumed 50–300 mg EGb 761/kg/day for 2–3 weeks (Blecharz-Klin et al., 2009; Yoshitake et al., 2010; Kehr et al., 2012). EGb 761 also has a slight inhibitory effect on acetylocholinesterase and therefore will increase cholinergic transmission in the brain (Zhang et al., 2018). It is believed that the main active substances responsible for improving cognitive functions are 1) ginkgolide, an inhibitor of platelet activating factor, inhibiting thrombocyte aggregation and improving blood circulation (Kidd, 1999; Lisiecka et al., 2016; Chen et al., 2019b; Goschorska et al., 2020), 2) bilobalide, increasing the expression of the glucocorticoid receptor in the hippocampus (Ma et al., 2012), observed in mice fed EGb 761 (2.5, 5 and 10 mg/kg/day) producing a decrease in the level of fear-like behavior and an increase in the motor activity of the tested animals in the open field and in a maze (Ma et al., 2012). Administration of bilobalide to mice for 4–8 weeks at 100 mg/kg/day (Winter 1991) and for 1–3 weeks at 40 mg/kg/day (Cohen-Salomon et al., 1997) improved memory in both young and aging animals. Bilobalide reduced cytotoxic brain edema in triethylin-induced as well as ischemic brain damage in the animals. Bilobalide prevented the hypoxia of organs and tissues, as well as the activation of phospholipase A2 and the degradation of phospholipids in the hippocampus of rats (Bodalski and Karłowicz-Bodalska, 2006), and protected against age-related changes in the mouse hippocampus (Ribeiro et al., 2016).

**TABLE 1:** Effect of *G. biloba* extracts EGb 761 on cognitive functions.

Number of subjects	Age (average)	Dose/treatment time	The effect obtained	References
Healthy volunteers	19.9	Standardized extract *G. biloba* (GK501) 120, 240 and 320 mg single doses, once a day	Better cognitive performance	Scholey and Kennedy (2002)
Healthy volunteers - 20	21.2	Standardized extract *G. biloba* (GK501) 360 mg single dose	Improvement of cognitive abilities, self-esteem and mood	Kennedy et al. (2002)
Healthy volunteers - 20 (study 1) - 28 (study 2) - 30 (study 3)	19.9 (study 1) 20.4 (study 2) 21.68 (study 3)	Standardized extract *G. biloba* (GBE) 120 mg once daily	Increasing attention efficiency, improving “memory quality”. The effect was most pronounced at 1 and 4 h post-dose	Kennedy et al. (2007)
Healthy volunteers/students - 52	18–26	Standardized extract *G. biloba* (LI1370) 120 mg/day for 6 weeks	Increased performance in cognitive tasks related to memory and attention maintenance, as found in pattern recognition. The effect was visible 4 hours after dosing	Elsabagh et al. (2005)
Patients with dementia - 222	>55	EGb 761 at 240 mg once daily for 24 weeks	Improvement of cognitive functions	Kanovski and Hoerr (2003)
Patients with mild to moderate dementia related to neuropsychiatric symptoms (AD or vascular dementia) - 410	>50	EGb 761 at 240 mg once daily for 24 weeks	Improvement in cognition, psychopathology, functional measurements and quality of life	Herrschaft et al. (2012)
Patients with mild to moderate dementia AD, vascular dementia, or mixed character - 410	>50	EGb 761 at 240 mg once daily for 24 weeks	Improvement of cognitive abilities, neuropsychiatric symptoms, functional capacity and general condition	Ihl et al. (2011)
Patients with mild to moderate dementia with neuropsychiatric features - 400	>50	EGb 761 at 240 mg once daily for 22 weeks	Overall improvement of cognitive functions	Napryeyenko and Borzenko (2007)
Patient with AD	>50	EGb 761, 240 mg/day for at least 22 weeks	Overall improvement of cognitive functions	Napryeyenko et al. (2009)
Elderly people with normal cognition or mild cognitive impairment - 3,069	72–96	*G. biloba* extract 120 mg twice a day	No significant change in cognitive function	Snitz et al. (2009)
People with very mild cognitive impairment - 300	45–65	EGb 761 at 240 mg once daily for 12 weeks	Improvement of cognitive functions, concentration as well as memory tasks related to everyday life and quality of life	Grass-Kanapke et al. (2011)
Patients with mild to moderate dementia - 25	50–80	EGb 761 at 160 mg once daily for 24 weeks	There was no significant difference between EGb 761 and donepezil in the treatment of mild to moderate dementia	Mazza et al. (2006)
Patients with cognitive impairment and dementia	25–61	EGb 761 at 240 mg once daily for 22–26 weeks	Stabilized or slowed decline in cognition function	Tan *et al. (2015)*
Patients with cognitive impairment and dementia - 60	50–65	EGb 761 at 240 mg once a day for 56 ± 4 days	Greater cognitive flexibility without altering brain activity in the elderly	Beck et al, (2016)
Patients with AD - 400	>50	EGb 761 at 240 mg once daily for 22 weeks	Less apathy and indifference, reduced anxiety, depression and irritability, and improved sleep	Scripnikov et al. (2007)
Healthy elderly or with mild cognitive impairment -3,069	>75	*G. biloba* extracts at 120 mg twice daily	EGb 761 was no better than placebo in reducing the rate of dementia progression in both elderly and AD patients	DeKosky et al. (2008)

## Safety in the Use of *G. biloba* Extract

### Application

Due to its broad spectrum of activity, preparations with *G. biloba* are used frequently, with the most popular being EGb 761 comprising a precisely defined chemical composition (Belwal et al., 2019; Lidner et al., 2019). The recommended dosage is 240 mg once daily (Kanovski and Hoerr, 2003; Napryeyenko et al., 2009; Tan et al., 2015; Thancharoen et al., 2019), or 120 mg twice daily (Elsabagh et al., 2005; Kennedy et al., 2007; DeKosky et al., 2008), and less frequently 320 mg once a day (Scholey and Kennedy, 2002).

### Interactions and Toxicity

Supplements with *G. biloba* may extend the bleeding and clotting time, which is exacerbated by the simultaneous administration of anticoagulants and antiplatelet drugs (Köhler et al., 2004). Kim et al. (2010) report that *G. biloba* extract can be administered simultaneously with some antiplatelet drugs, such as ticlopidine, which the authors of the study used with 24 healthy volunteers at a single dose of 80 mg, combined with a 250 mg extract of *G. biloba*. The use of both preparations together did not extend the bleeding time compared to the administration of ticlopidine alone (Kim et al., 2010). A sudden increase in blood pressure may occur with diuretics. The extract may also enhance the effect of antidepressants such as phenelzine, tranylcypromine and trazodone, causing an increased risk of side effects and even a coma. When *G. biloba* is taken simultaneously and for a long time with non-steroidal anti-inflammatory drugs, such as ibuprofen, diclofenac or ketoprofen, an irritating effect on the gastric mucosa may intensify, leading to ulcers and gastrointestinal bleeding (Abebe, 2002). On the other hand, drugs used for gastric ulcers, such as omeprazole, show a reduced effect in the presence of the extract of *G. biloba* (Bojarowicz and Dźwigulska, 2012). *G. biloba* also has a blood sugar elevating effect, so in diabetic patients taking the extract, blood glucose monitoring is recommended (Xin et al., 2014). *G. biloba* extract affects the biotransformation of drugs by affecting the activity of cytochrome P450. The terpenoid fraction contained in the plant inhibits cytochrome CYP2C9, while the flavonoid fraction reduces the activity of cytochromes CYP2C9, CYP1A2, CYP2E1 and CYP3A4. In addition, studies have shown the effect of *G. biloba* and single substances isolated from it on the activity of P-glycoprotein (Bogacz et al., 2013; Rogowska and Ciermaziak, 2018). Li et al. (2009) showed that ginkgolide A and B induce hepatic P-glycoprotein, as well as a decrease in the metabolism of nicardipine, a calcium channel blocker. Such an effect may reduce the rate of drug metabolism due to inhibition of CYP3A, reducing its antihypertensive effect (Yoshioka et al., 2004), while a single dose of *G. biloba* did not affect the pharmacokinetics of talinolol, but repeated administration of the extract maximized the plasma concentration of the drug by 36% (Fan et al., 2009). Markowitz reports that the *G. biloba* extract in the recommended doses probably does not change the effect of co-administered drugs dependent on the CYP2D6 or CYP3A4 elimination pathways, such as dextromethorphan and alprazolam, which were used at a dose of 120 mg twice a day together with *G. biloba* extract (Markowitz et al., 2003).

In general, the EGb 761 extract is well tolerated and safe up to a dose of 240 mg/day (Ahlemeyer and Krieglstein, 2003; Kalisz et al., 2006; Hashiguchi et al., 2015; Awortewe et al., 2019). No side effects were observed when *G. biloba* extract was administered for 1–3 months at a dose of 120–160 mg/day (Kleijnen and Knipschild, 1992). In contrast, in a similar study at a dose of 120 mg/day for 52 weeks, gastrointestinal complications were common (Le Bars et al., 1997). The main adverse reaction reported by patients with AD during EGb 761 treatment was a headache (Ihl et al., 2011). It is generally believed that the use of *G. biloba* does not show strong side effects, but if they do occur they are mainly: nausea, vomiting, diarrhea, headache and dizziness, weakness, skin rashes, palpitations (Mahadevan and Park, 2007). So far, no mutagenic, carcinogenic, teratogenic or embryogenic side effects have been demonstrated. The oral LD50 in mice is 2,100 times the recommended daily dose (Smith and Luo, 2003).

EGb 761 contains allergenic and toxic compounds such as 4-O-methylpyridoxin (ginkgotoxin) (Leistner and Drewke, 2010), so the concentration in EGb 761 is limited to 5 ppm (Abdel-Kader et al., 2007). Ginkgotoxin is found mainly in the seeds and leaves of *G. biloba* and is structurally related to vitamin B6, possibly interfering with its synthesis, metabolism and functioning. Excessive consumption of ginkgotoxin may cause seizures (Kästner et al., 2007; Belwal et al., 2019). Ingestion of the seeds can be poisonous, leading to severe allergic skin reactions as well as seizures (Hasegawa et al., 2006; Pennisi, 2006). Possible nephrotoxicity and hepatotoxicity of other bioflavonoids in *G. biloba* leaves, such as amentoflavone, sciadopitysin, ginkgetin, isoginkgetin and bilobetin, have also been demonstrated. In an *in vitro* cytotoxicity test, these compounds reduced cell viability in human tubular renal epithelial cells (HK-2) and human normal hepatocytes (L-02), indicating potential hepatic and renal toxicity. In contrast, in the *in vivo* experiment, phosphatase activity was significantly elevated after mice ingested these substances. Widespread hydropic degeneration of hepatocytes was observed after administration of ginkgetin and bilobetin (Li et al., 2019).

The undoubted advantage of using *G. biloba* derivatives is indicated by reports on the safety of this drug. Comparative studies of EGb 761 and donepezil do not show significant differences in the effectiveness of either substance (Mazza et al., 2006). In a study involving older patients (80 and older), it was found that EGb 761 showed similar procognitive effects to donepezil, while presenting fewer side effects (Rapp et al., 2018).

## Perspectives on New Research

Multipotent substances acting at many different levels with increased efficacy, a safe profile and an easy administration route in the treatment of AD, are currently being sought. Recent studies often mention the docking method, which is widely used in the design of multipotent drugs (Li et al., 2018). The pathogenesis of AD is complex and multifactorial, hence the goal of pharmacotherapy should be to influence a number of proteins and enzymes, including NMDA, nitric oxide synthase (NOS), beta secretase 1 (BACE-1), TNFα, mono amine oxidase A (MAO-A), mono amine oxidase B (MAO-B) and butylcholine esterase (Grewal et al., 2020). Computer models prove the effectiveness of drug selection based on the docking method, especially in the field of the phenolic derivatives contained in EGb 761 and pharmacological targets in AD (Grewal et al., 2020). Even in 2009, meta-analyses showed inconclusive results in research on the effectiveness of *G. biloba* (Birks and Evans, 2009). In the latest reports from a metanalysis of research from 1980 to 2020, information is found that EGb 761 may be able to improve cognitive functions in patients suffering from mild dementia while using the drug for a long period of time–more than 24 weeks at an appropriate dose of 240 mg/day (Liu et al., 2019). Most authors agree that more extensive randomized trials with varying doses and long follow-up periods are needed.

## Conclusion

*G. biloba* derivatives have a proven beneficial effect on cognitive disorders in the course of dementia, including mild and moderate AD. Unfortunately, there are not many randomized trials proving the clinical effectiveness in AD. Despite its recognized position in the treatment of neurodegenerative diseases, there is still controversy over the exact mechanism of its action, as well as its clinical effectiveness and application. It seems reasonable to assume that the effect of *G. biloba* derivatives will be individual and will depend on the duration of the treatment as well as the applied dose. So far, no limitations on the use of ginkgo derivatives have been identified. Perhaps one of the factors determining the effectiveness of a treatment is the patient’s sensitivity to a given drug (homogeneity, heterogeneity of the population). There is also a need for extensive research depending on the severity of the course, genetic condition and dose used in treatment, with long-term follow-ups.
